# Rethinking ejection fraction for anesthesiologists: a narrative review

**DOI:** 10.1186/s13741-026-00690-5

**Published:** 2026-05-06

**Authors:** Marco Rabis, Patrick Moldzio

**Affiliations:** 1 Department of Anesthesiology, Montefiore Medical Center, Albert Einstein College of Medicine, Bronx, NY USA; 2 Department of Anesthesiology, Intensive Care, and Pain Therapy, Marienhospital Bottrop – Knappschaft Kliniken, Bottrop, Germany

**Keywords:** Ejection fraction, Perioperative risk stratification, Heart failure phenotypes, Physiology-guided anesthesia, Perioperative echocardiography, Global longitudinal strain, Biomarker- integrated assessment

## Abstract

Left ventricular ejection fraction (EF) is one of the most frequently cited cardiac parameters in perioperative risk assessment and is often used as a surrogate marker of global cardiac stability. However, EF was validated primarily in chronic cardiovascular disease and long-term heart failure management, raising important questions about its suitability as a dominant marker of short-term perioperative risk. EF does not capture key determinants of perioperative vulnerability, including diastolic function, ventricular compliance, right ventricular function, venous congestion, or contractile reserve. Contemporary clinical data suggest that only severely reduced EF (< 30%) is consistently associated with markedly increased perioperative risk, whereas mildly to moderately reduced EF in clinically stable patients often carries less prognostic significance than preserved EF in the presence of heart failure with preserved ejection fraction (HFpEF), congestion, or right ventricular dysfunction. Common perioperative scenarios such as HFpEF, distributive shock, hypovolemia, venous congestion, and chronic compensated heart failure illustrate that EF may substantially underestimate or overestimate true hemodynamic vulnerability depending on the physiological context. In contrast, functional capacity, volume status, and clinical stability emerge as more robust determinants of short-term perioperative risk. Advanced echocardiographic parameters, including diastolic indices, global longitudinal strain (GLS), and focused assessment of right ventricular function, together with biomarkers provide a more physiologically coherent risk profile. The uncritical use of EF as a primary perioperative risk marker is not supported by current evidence. For anesthesiologists, EF should be interpreted as one component within a multidimensional, physiology-guided assessment that integrates focused perioperative ultrasound, clinical history, and targeted biomarkers to more accurately characterize perioperative cardiovascular risk.

## Introduction

Over the past three decades, echocardiography has evolved from a cardiology-centered diagnostic modality into a core clinical skill within anesthesiology. It is encompassing perioperative transesophageal echocardiography (TEE), transthoracic echocardiography (TTE), and focused point-of-care ultrasound. The number of echocardiographic examinations performed by anesthesiologists continues to increase (Subramaniam et al. 2021; Shillcutt et al. 2017; Barber and Fletcher 2014). This development has been accompanied by structured training pathways, formalized curricula, and national and international certification programs defining competency in perioperative echocardiography. Within this expanding scope of practice, left ventricular ejection fraction (EF) has assumed a central role as one of the most frequently reported and interpreted echocardiographic parameters. EF is often used to communicate “cardiac risk” in the perioperative setting. This concept originates from early validation studies of two-dimensional echocardiography (Schiller et al. 1979). Its central position is most evident in the field of heart failure. Contemporary international consensus statements designate EF as the primary criterion for classifying heart failure (HF) into reduced (HFrEF), mildly reduced (HFmrEF), and preserved (HFpEF) subtypes (Rosano et al. 2025). These categories directly determine eligibility for evidence-based pharmacologic therapies, device implantation, and long-term prognostic assessment. No alternative imaging or biomarker-based parameter has replaced EF in this role (Rosano et al. 2025; Palazzuoli et al. 2025).

Consequently, EF has also become the most prominent cardiovascular reference parameter in research. Its predominance is rooted in the 2004 recommendations of the American Society of Echocardiography (ASE), which defined methodological standards for the use of echocardiography in clinical trials (Gottdiener et al. 2004). These guidelines have shaped inclusion criteria for heart failure and post-myocardial infarction studies for more than two decades. However, the document has not been updated since its publication, and contemporary imaging modalities and pathophysiological concepts are not reflected in its recommendations.

In parallel, the central role of EF in cardiology has become increasingly debated in recent literature (Dell'Angela and Nicolosi 2024). There is growing recognition of its limitations and potential alternatives that may offer superior sensitivity or prognostic value (Dell'Angela and Nicolosi 2024; Halliday et al. 2021; Lisi et al. 2024). Despite these limitations, EF continues to function as a research standard.

While EF is firmly established in chronic cardiovascular care and long-term clinical decision-making, the perioperative setting represents a different physiological and temporal context. This narrative review provides a targeted, non-systematic synthesis of current evidence with a focus on physiological interpretation and perioperative decision-making.

In routine anesthetic assessment, EF is often treated as a defining marker of cardiac stability, and its presence in the medical record typically conveys an immediate, though not always accurate, impression of perioperative risk. A contemporary review on perioperative cardiovascular risk assessment suggests that EF is primarily relevant only at the extreme end of systolic dysfunction (Smilowitz and Berger 2020). In the large cohort underpinning these recommendations, only severely reduced EF (< 30%) was clearly associated with a markedly increased risk of major adverse cardiovascular events (MACE) (Smilowitz and Berger 2020). The discrepancy between its established role in chronic cardiology and its unvalidated application in perioperative evaluation underscores the need to re-evaluate its utility and to identify parameters better aligned with perioperative physiology.

## Physiological limitations of Ejection Fraction

**Fig. 1:**
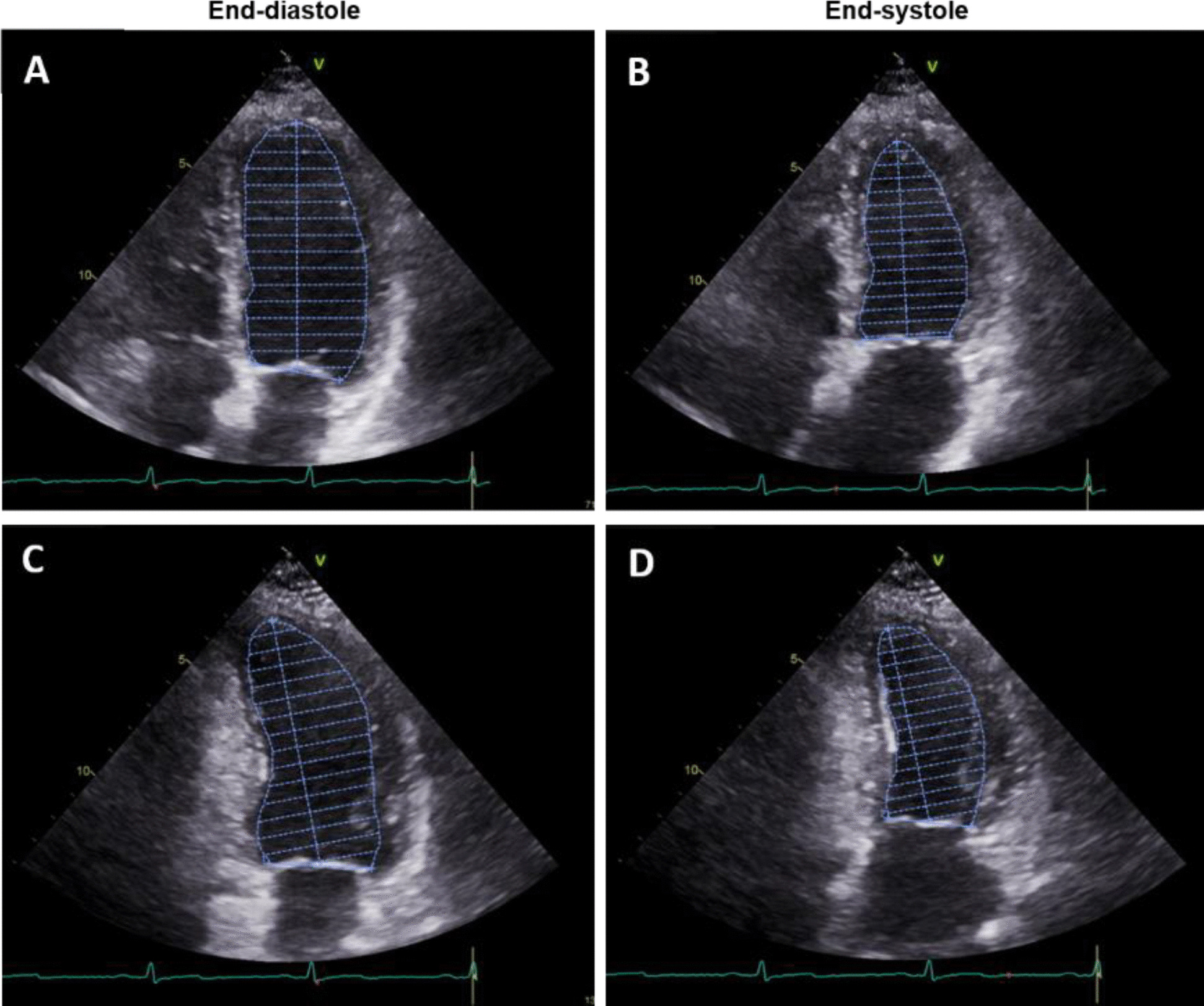
Assessment of left ventricular EF using modified Simpson method. Apical four-chamber (**A**,**B**) and two-chamber (**C**,**D**) transthoracic echocardiographic views in end-diastole (**A**,**C**) and end-systole (**B**,**D**) illustrating the biplane method of disks (modified Simpson’s method) for the assessment of left ventricular ejection fraction. Reproduced with permission from Scatteia A. et al., under the terms of the Creative Commons Attribution License (CC BY 4.0) (Scatteia, et al., 2021)

EF quantifies the fraction of end-diastolic volume ejected per beat and is typically derived from two-dimensional biplane imaging (modified Simpson method, Fig. 1). Despite its familiarity, EF is an image-quality-dependent estimate of global systolic function, highly sensitive to prevailing preload and afterload. In perioperative patients, loading conditions are continuously altered by anesthetic induction, mechanical ventilation, vasoactive drugs, positioning, bleeding, and fluid shifts. These determinants are not captured by a preoperative, resting EF value.

Importantly, EF reduces complex cardiovascular function into a single static metric. EF does not distinguish between stroke volume and effective forward flow and therefore does not reliably reflect tissue perfusion. Furthermore, it does not characterize diastolic function, right heart physiology, or contractile reserve. EF may appear preserved or even supranormal in vasodilatory or hypovolemic states despite profound circulatory failure. On the other hand, chronically reduced EF may coexist with stable hemodynamics in compensated patients on optimized therapy. Small differences around commonly cited cutoffs may reflect measurement variability rather than true changes in physiological reserve.

## Clinical scenarios where EF misleads

While some of the following evidence is derived from critical care or non-perioperative settings, these data were used to illustrate fundamental physiological principles that directly relevant to perioperative hemodynamic management.

### Heart Failure with Preserved Ejection Fraction (HFpEF)

HFpEF represents one of the most clinically relevant scenarios in which EF fails to reflect perioperative cardiovascular vulnerability. Despite preserved systolic function patients with HFpEF frequently exhibit impaired ventricular relaxation, increased chamber stiffness, elevated filling pressures. These pathophysiological features render them particularly susceptible to perioperative pulmonary congestion, myocardial injury, and hemodynamic instability (Pagel et al. 2021). Despite the fact that general anesthesia tends to reduce preload, afterload, and may lower heart rate, they do not enhance intrinsic diastolic function (Couture et al. 2009). Intraoperative studies have shown that while cardiac output (CO) may be maintained, EF can decrease due to increased end-diastolic volume (Brinke et al. 2008).

Nevertheless, HFpEF is a significant independent risk factor for increased perioperative mortality in patients undergoing non-cardiac surgery. According to the most recent guideline from the American College of Cardiology (ACC) and the American Heart Association (AHA), patients with HFpEF have a 90-day mortality rate of 4.88% after non-cardiac surgery, compared to 1.22% in patients without heart failure. The adjusted odds ratio for 90-day mortality in HFpEF is 1.51 (95% CI 1.40—1.62), indicating a substantial increase in risk (Thompson et al. 2024). HFpEF patients experience nearly double the adjusted odds of postoperative mortality compared to those without heart failure (Lerman et al. 2019).

**Fig. 2:**
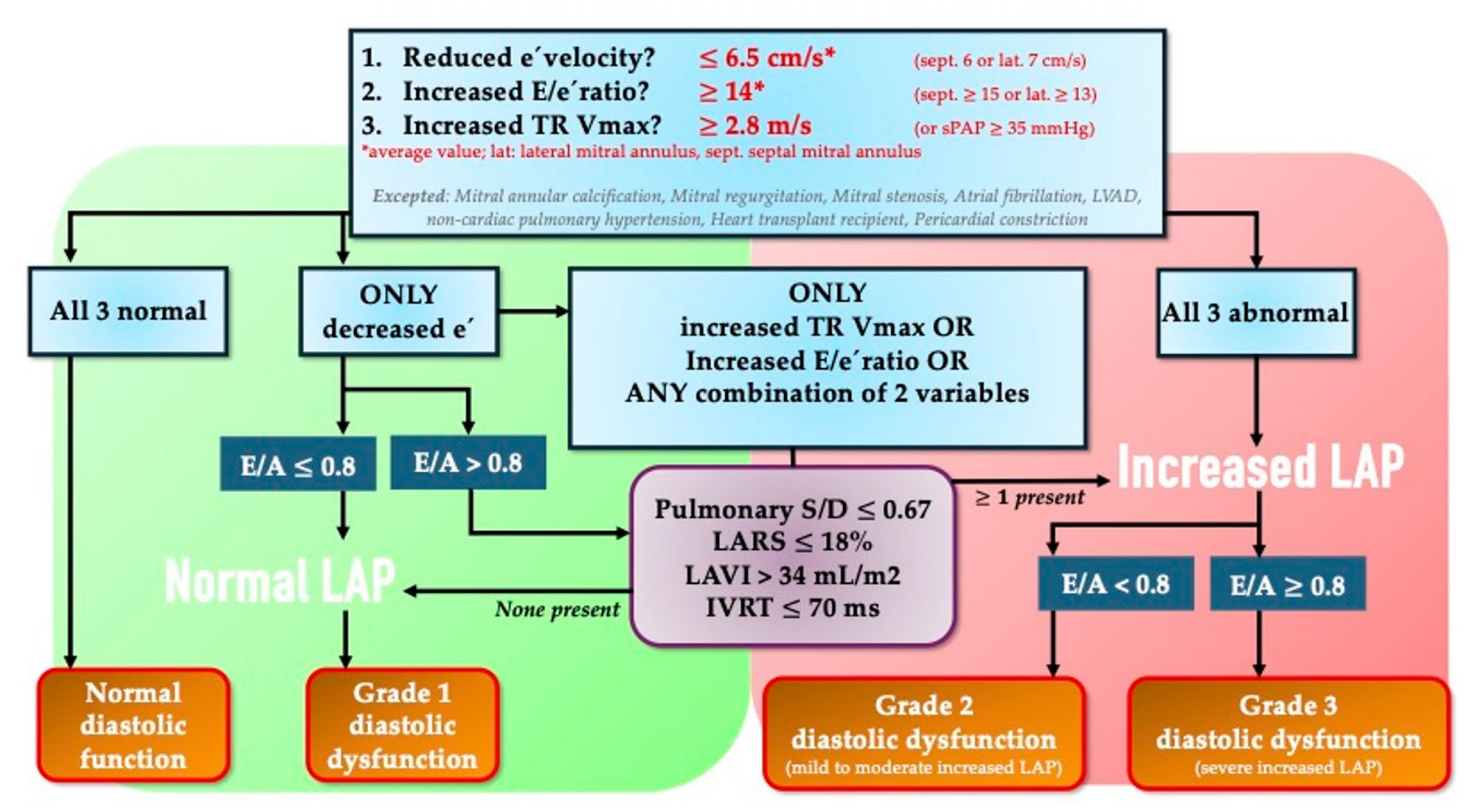
2025 ASE algorithm for the echocardiographic diagnosis and grading of left ventricular diastolic dysfunction and estimation of LAP. Diagnostic approach is based on 3 key parameters: e´ mitral annular velocity, E/e´ ratio, and TR Vmax. Additional parameters, including the E/A ratio, pulmonary vein systolic/diastolic (S/D) ratio, LAVI, LARS, and IVRT, are used to refine classification in intermediate cases (reproduced from Nagueh et al., under the terms of CC BY 4.0) (Nagueh et al. 2025). ASE: American Society of Echocardiography; IVRT: Isovolumetric relaxation time; LAP: Left atrial pressure; LARS: Left atrial reservoir strain; TR Vmax: Peak tricuspid regurgitation velocity

In contrast to HFrEF, HFpEF is not an entity that is immediately apparent on cardiac imaging but requires targeted evaluation. Contemporary echocardiography provides a structured and well-validated diagnostic approach, as outlines in the 2025 ASE recommendations (Fig. 2) (Nagueh et al. 2025).

### Mitral Regurgitation

Mitral regurgitation (MR) represents a common clinical scenario in which EF may overestimate left ventricular function. EF cannot distinguish between effective forward flow into the aorta and regurgitant flow into the low-pressure left atrium. As a result, EF may appear normal despite reduced effective cardiac output (Bonow et al. 2015). This discrepancy becomes particularly relevant in the perioperative setting, where changes in load or acute volume shifts can unmask latent ventricular dysfunction and precipitate hemodynamic instability. (Thompson et al. 2024; Bonow et al. 2015). Importantly, this limitation is not confined to severe MR but may already be clinically relevant in moderate disease (Hussein et al. 2017)

### Distributive shock and hypovolemia

In distributive shock or hypovolemic states, often studies in critical care settings but highly relevant as models of altered loading conditions, EF may appear preserved or even supranormal. This reflects reduced afterload rather than actual cardiac function. Among surgical ICU populations, hospital mortality for septic shock is consistently reported around 37—39%, and mortality rates are even higher in patients with persistent organ dysfunction (Vincent et al. 2019; Cecconi et al. 2018; Brakenridge et al. 2019). While Dugar et al. showed that both, patients with severely reduced EF (< 30%) and hyperdynamic EF (> 70%), are independently associated with increased in hospital mortality (Dugar et al. 2023). Mild to moderate reduction in EF do not consistently predict worse outcomes in septic shock, and acute changes in EF during sepsis are not automatically associated with higher mortality. (Forner et al. 2024; Chotalia et al. 2022).

In hypovolemic states, reported mortality rates reach approximately 46%, with most deaths attributable to ongoing hemorrhage (Harvin et al. 2017). Hypovolemia is also, frequently accompanied by a hyperdynamic circulatory state. It is driven by reduced afterload and compensatory sympathetic activation. Consistently with this physiology, observational data indicate that supranormal EF is associated with increased mortality (Chotalia et al. 2022). These Patients may exhibit profound circulatory failure despite preserved or supranormal EF values.

### Venous congestion and volume status

Venous congestion is increasingly recognized as a key determinant of perioperative morbidity, particularly with respect to pulmonary edema, acute kidney injury, and postoperative delirium. Venous congestion is typically quantified by an elevated central venous pressure (CVP). An increased CVP may reflect elevated hydrostatic pressure and a thereby promoted interstitial edema. Increased hydrostatic pressure in the lungs could lead to pulmonary edema and barrier dysfunction, which can trigger local and systemic organ injury (Husain-Syed et al. 2015). In the kidneys, venous congestion reduces renal perfusion pressure and promotes renal edema, resulting in a higher risk of AKI. As observational studies have shown, each 10-min period of intraoperative CVP ≥ 12 mmHg increases the hazard for acute kidney injury (AKI), and CVP above 14 mmHg doubles the risk of AKI after cardiac surgery (Gambardella et al. 2016). In a retrospective cohort of 12,778 patients, Chen et al. demonstrated that peripheral edema and elevated CVP were independently associated with a 30% increased risk of AKI. Consistently, intraoperative venous congestion (CVP ≥ 12 mmHg) is frequent during cardiac surgery and is accompanied by AKI rates of 20–25% (Chen et al. 2022; Lopez et al. 2021; Li et al. 2024).

Venous congestion also contributes to postoperative delirium, likely through cerebral edema and inflammation associated with fluid overload (Mosharaf et al. 2025). There is only few data, but cumulative fluid imbalance is associated with increased odds of delirium after cardiac surgery with an OR of 1.20 per liter positive balance (Mailhot et al. 2019). Given this, EF alone does not capture central venous pressure or right-sided pressures as critically influences of organ perfusion and postoperative outcomes. Clinically, venous congestion is highly relevant, as it is independently associated with increased postoperative mortality, morbidity, and longer hospital stays (Husain-Syed et al. 2015; Gambardella et al. 2016; Chen et al. 2022; Mailhot et al. 2019; Chen et al. 2016). In the perioperative context, assessment of volume status and venous congestion is often more informative than EF measurements, especially when anticipating fluid responsiveness, pulmonary complications, or renal vulnerability (Deschamps et al. 2023).

### Chronic, compensated heart failure

EF does not reliably detect subclinical myocardial injury, persistent fibrosis, or ongoing neurohormonal activation. This is particularly relevant in patients with chronic, compensated heart failure. These patients may exhibit stable perioperative courses when adequately compensated and treated with guideline-directed medical therapy (GDMT) even with severely reduced EF. In these cases, EF alone may overestimate perioperative risk, particularly when interpreted without consideration of functional status, volume balance, and clinical stability (Marwick 2018; Kodur and Tang 2025).

GDMT, including beta-blockers, angiotensin receptor-neprilysin inhibitors (ARNI), mineralocorticoid receptor antagonists, and SGLT2-inhibitors, has been shown to induce left ventricular remodeling. They also improve EF, and reduce morbidity and mortality across the whole spectrum of heart failure phenotypes (Riccardi et al. 2025; Dimond et al. 2024). Patients with recovered or improved EF have lower perioperative risk than those with persistent HFrEF. The time course of improvement and the completeness of GDMT are the more critical determinants of perioperative risk (Dimond et al. 2024; Wilcox et al. 2020).

## Better predictors of perioperative risk

### Role of functional status, volume balance, and clinical stability

Functional status, volume management, and clinical stability are essential components of perioperative risk assessment and often supersede EF alone. The ACC and AHA emphasize that perioperative risk is driven by symptoms, evidence of congestion, and neurohormonal activation, regardless of EF (Thompson et al. 2024; Pagnesi et al. 2023). Patients who are euvolemic, physically active, and clinically stable on GDMT have a substantially lower risk of perioperative complications than those with decompensated heart failure, even if their EF remains reduced. Congestion and volume overload are key determinants of adverse outcomes, and perioperative volume management is paramount (Pagnesi et al. 2023).

Beyond imaging-based assessments, no single parameter can substitute for clinical judgment when interpreting symptoms, disease trajectory, and current stability. Within this framework, validated risk scores provide valuable support. The Revised Cardiac Risk Index (RCRI) is the best validated and most widely used tool for estimating perioperative cardiovascular risk in patients undergoing noncardiac surgery to estimate the likelihood of MACE and is therefore recommended by the ACC/AHA (Smilowitz and Berger 2020; Thompson et al. 2024; Lee et al. 1999).

### Integration into established perioperative risk frameworks

Contemporary perioperative risk assessment follows structured, guideline-based algorithms, most prominently reflected in the ACC/AHA recommendations (Thompson et al. 2024). These frameworks emphasize a selective, symptom-driven approach rather than routine imaging-based screening.

These recommendations highlight that echocardiographic assessment of left ventricular function is applied selectively and within a broader clinical context rather than a routine screening tool. In this way EF is not used as a primary determinant of perioperative risk but rather contributes to the broader characterization of underlying cardiac disease. Key elements of perioperative risk stratification include the presence of active cardiac conditions, functional capacity, and overall clinical stability. Validated tools such as the RCRI remain central to this process (Lee et al. 1999).

When EF is assessed, its prognostic value is context dependent. Severely reduced EF (≤ 30%) is consistently associated with increased perioperative risk, particularly in symptomatic patients undergoing higher-risk procedures (Thompson et al. 2024). However, across a broader range of EF values, risk discrimination remains limited, and functional capacity provides independent and often stronger prognostic information (Smilowitz and Berger 2020).

### Advanced imaging

Growing evidence has highlighted the limitations of EF in capturing myocardial mechanics and stress tolerance (Marwick 2018). In the perioperative context, echocardiography-based strain analysis, measured by speckle-tracking echocardiography, has emerged as a particularly practical and informative modality.

Particularly global longitudinal strain (GLS) is an advanced echocardiographic technique bridging advanced myocardial assessment with bedside applicability. It quantifies myocardial deformation and provides sensitive, reproducible assessment of both global and regional cardiac function. Unlike EF, strain imaging detects subclinical systolic dysfunction, is less dependent on loading conditions, and does not rely on geometric assumptions. This makes GLS a more robust marker of myocardial health in the perioperative setting (Benson et al. 2020; Smiseth et al. 2016). An impaired GLS is independently associated with adverse outcomes (Table 1), including low cardiac output states (LCOS), need for inotropic support, postoperative arrhythmias, and increased length of hospital stay (Smiseth et al. 2016; Abuelkasem et al. 2019; Mihos et al. 2025).

**Table 1:** Guideline-based indications for preoperative assessment of left ventricular function

*Recommendation Class*	*Clinical Scenario*	*Clinical Implication*
Class I (LV function assessment is recommended)	Patients with new dyspnea, physical examination findings of heart failure, or suspected new/worsening ventricular dysfunction	Identify previously undiagnosed or unstable cardiac pathology, and guide perioperative management
Class II (LV function assessment is reasonable)	Patients with known heart failure and worsening symptoms or change in clinical status	Supports risk stratification and optimization of HF therapy prior to surgery
Class III (LV function assessment is not recommended)	Asymptomatic and clinically stable patients undergoing noncardiac surgery	Avoids unnecessary imaging without proven benefit for perioperative outcomes

Guideline-based recommendations for preoperative assessment of left ventricular (LV) function according to the 2024 AHA/ACC perioperative cardiovascular management guideline. (Thompson et al. 2024) Recommendations emphasize a selective, symptom-driven approach rather than routine echocardiographic screening in clinically stable patients

In a cohort of cardiac surgery patients, Ternacle et al. found abnormal GLS in approximately 40% of patients with preserved EF, indicating that strain analysis may allow earlier identification of myocardial vulnerability and maybe improved prediction of adverse perioperative outcomes. Mentias et al. demonstrated that a GLS > −21% was associated with increased risk and long-term mortality despite preserved EF in patients undergoing mitral valve repair (Mentias et al. 2016).

The ASE and the European Association of Cardiovascular Imaging (EACVI) recommend strain imaging for nuanced risk stratification and individualized perioperative management (Thomas et al. 2025). Integration of strain imaging into perioperative echocardiography enhances the ability to tailor fluid strategies, optimize timing of interventions, and improve patient outcomes (Benson et al. 2020; Abuelkasem et al. 2019; Mihos et al. 2025; Mentias et al. 2016; Thomas et al. 2025).

### Biomarkers, and individualized risk assessment

Circulating biomarkers provide complementary information to imaging-based parameters by reflecting ongoing myocardial injury and end-organ vulnerability. Natriuretic peptides, particularly NT-proBNP, integrate multiple pathophysiological processes, including myocardial wall stress, volume overload, and neurohormonal activation. They are consistently associated with perioperative cardiovascular complications. Elevated preoperative NT-proBNP identifies patients at increased risk for heart failure exacerbation, myocardial injury, and postoperative mortality, even in the absence of overt systolic dysfunction (Table 2) (Clerico et al. 2022).

**Table 2:** Perioperative relevance of myocardial strain parameters

*Parameter*	*Normal range*	*Pathological threshold*	*Perioperative relevance*
Global Longitudinal Strain (GLS)	≤ − 17%	> − 18%	Predictor of MINS (Kim et al. 2023)
		> − 17%	Increased risk for LCOS (Amabili et al. 2018)
		> − 17% > − 16%	Subclinical systolic dysfunction; ~ 40% abnormal GLS in patients with normal EF (Ternacle et al. 2013) Improves discriminatory power of EuroSCORE II (Ternacle et al. 2013)
		> − 21%	Increased perioperative risk and mortality in patients undergoing MVR, regardless of EF (Mentias et al. 2016)
Left Atrial Reservoir Strain (LARS)	> 27.5%	< 20%	Increased risk of postoperative AF (Granchietti et al. 2025)
		< 20%	Increased risk of MACE (Tan et al. 2023)
		< 18%	Diagnostic criterion of LV-DD (Nagueh et al. 2025)

This table summarizes normal ranges, pathological thresholds, and clinical relevance of strain parameters. *Strain values are expressed as absolute percentages; less negative GLS values indicate impaired myocardial deformation*

*AF* Atrial fibrillation, *ASE* American Society of Echocardiography, *EF* Ejection fraction, *GLS* Global Longitudinal Strain, *LARS* Left Atrial Reservoir Strain, *LCOS* Low Cardiac Output Syndrome, *LV-DD* Left ventricular diastolic dysfunction, *MACE* Major adverse cardiovascular events, *MINS* Myocardial injury after non-cardiac surgery, *MVR* Mitral valve repair

Cardiac troponins serve as sensitive markers of myocardial injury and have relevance in the perioperative setting (Table 2). Even minor elevations are associated with adverse short- and long-term outcomes (Writing Committee for the VISION Study Investigators 2017). Preoperative troponin elevation may indicate subclinical myocardial vulnerability, while postoperative increases frequently reflect myocardial injury after noncardiac surgery (MINS) (Shen et al. 2018; Maile et al. 2016). Importantly, troponin abnormalities often occur independently of EF or may identify high-risk patients who would otherwise be misclassified as low risk (Januzzi et al. 2019; Tveit et al. 2023).

## Implications for anesthesiologists

Despite its limitations, EF retains perioperative relevance in selected clinical contexts. Severely reduced EF (< 30%) remains a strong marker of increased perioperative risk and warrants heightened vigilance (Thompson et al. 2024; Doherty et al. 2024). However, beyond the extremes, EF alone provides limited discrimination of short-term perioperative vulnerability and should be interpreted within a broader physiological framework (Tables 3 and 4).

**Table 3:** Perioperative prognostic thresholds of cardiac biomarkers

*Biomarker*	*Threshold*	*30-day mortality*
NT-proBNP (pre-OP) (Thompson et al. 2024; Doherty et al. 2024)	< 100 pg/mL	0.3%
	100—200 pg/mL	0.7%; HR for MACE 2.27
	200—1500 pg/mL	1.4%; HR for MACE 3.63
	> 1500 pg/mL	4.0%; HR for MACE 5.82
Troponin (hs-cTnI/hs-cTnT) (post-OP) (Thompson et al. 2024; Januzzi et al. 2019)	< 14 ng/L	0.01—0.05%
	14—20 ng/L	1.1%
	21—64 ng/L	3.0%
	65—999 ng/L	9.1%
	> 1000 ng/L	29.6%

This table summarizes perioperative thresholds of established cardiac biomarkers and their association with 30-day mortality and major adverse cardiovascular events in patients undergoing non-cardiac surgery

*HR* Hazard ratio, *hs-cTnI* high-sensitive cardiac troponin I, *hs-cTnT* high-sensitive cardiac troponin T, *MACE* Major adverse cardiac events, *NT-proBNP* N-terminal pro-B-type natriuretic peptide

**Table 4:** What should anesthesiologists focus on?

*Domain*	*What EF misses*	*Key perioperative assessments*	*Why it matters*
Functional capacity	Daily activity tolerance	METs, RCRI, NYHA class, recent HF admission, GDMT status	Predicts stress tolerance (Thompson et al. 2024; Doherty et al. 2024)
Volume state	Venous congestion	CVP, IVC, venous Doppler, lung US	AKI, pulmonary edema, delirium (Husain-Syed et al. 2015; Gambardella et al. 2016; Chen et al. 2022; Mailhot et al. 2019; Chen et al. 2016; Husain-Syed et al. 2018)
Contractile reserve	Stress response	GLS, NT-pro-BNP, troponin	Hypotension, MINS (Thompson et al. 2024; Ternacle et al. 2013; Doherty et al. 2024; Januzzi et al. 2019; Tveit et al. 2023; Borlaug et al. 2010)
RV & pulmonary pressures	RV afterload sensitivity	RV function, PAP	Fluid and Ventilation intolerance (Rajagopal et al. 2023; Houston et al. 2023)

This table summarizes clinical domains relevant to perioperative cardiovascular risk stratification

*EF* Ejection Fraction, *METs* Metabolic equivalents, *RCRI* Revised Cardiac Risk Index, *NYHA* New York Heart Association classification, *HF* Heart Failure, *GDMT* Guideline-directed medical therapy, *CVP* Central Venous Pressure, *IVC* Inferior Vena Cava diameter, *US* Ultrasound, *AKI* Acute Kidney Injury, *GLS* Global Longitudinal Strain, *MINS* Myocardial Injury after Non-cardiac Surgery, *RV* Right Ventricle, *PAP* Pulmonary Arterial Pressure

### Clinical implications for perioperative management

The limitations of EF in reflecting perioperative cardiovascular vulnerability have direct implications for clinical decision-making. Rather than relying on EF as a primary determinant, perioperative management should be guided by a multidimensional assessment integrating functional capacity, volume status, and biomarkers.

In the preoperative setting, functional capacity, particularly the inability to achieve < 4 metabolic equivalents (MET), and elevated natriuretic peptides identify patients at increased perioperative risk more reliably than EF alone (Smilowitz and Berger 2020; Thompson et al. 2024; Karthikeyan et al. 2009).

Intraoperative management should focus on dynamic assessment, including volume responsiveness, right ventricular function, and ventricular interdependence (Thompson et al. 2024; Rajagopal et al. 2023; Chaves et al. 2024). Focused echocardiography allows real-time evaluation of volume status, contractility, volume responsiveness. Thereby it enables individualized, physiology-guided hemodynamic management rather than decisions based on historical EF measurements.

Postoperatively, surveillance strategies should prioritize early detection of myocardial injury and decompensation. Cardia biomarkers, particularly troponin and natriuretic peptides, are sensitive markers of perioperative myocardial stress and are more closely associated with short-term outcomes than isolated EF measurements (Association Between Postoperative Troponin Levels and 30-Day Mortality Among Patients Undergoing Noncardiac Surgery 2012); Devereaux and Sessler 2015).

Taken together, these considerations support a multidimensional, physiology-guided approach to perioperative care that integrates clinical history, current physiological status, and biomarker assessment within a patient-centered framework. Where appropriate, focused echocardiography may further refine this assessment by providing real-time, bedside insights into cardiovascular function.

### Reframing EF and practical implications for the perioperative assessment

A patient with an EF of 28% who is clinically stable, euvolemic, and well controlled on GDMT may tolerate anesthesia more reliably than a patient with preserved EF and advanced diastolic dysfunction and/or venous congestion. Absolute EF values therefore do not directly translate into perioperative hemodynamic tolerance. Several clinically relevant aspects of cardiac function are not addressed by EF alone. These include filling pressures, venous congestion, ventricular compliance, diastolic reserve, right ventricular function, pulmonary pressures, and the ability of the myocardium to augment output under acute stress (Thompson et al. 2024). In addition, EF may change substantially during anesthesia. A resting EF value measured days or weeks before surgery therefore offers limited insight into real-time cardiovascular capacity during induction and intraoperative stress.

For anesthesiologists, the most pragmatic approach is to interpret EF within a physiological framework. This often requires reconciling patient history, previous imaging reports, and the current clinical picture. Focused physician-performed echocardiography plays a key role in this process by allowing the anesthesiologist to verify crucial clinical information at the point of care. The ability to perform such examinations strengthens, rather than replaces the value of EF. It embeds EF within the clinical reality rather than relying on historical documentation.

Severely reduced EF (< 30%) is associated with increased perioperative risk, particularly when accompanied by symptoms, congestion, or recent decompensation. However, mildly to moderately reduced EF in clinically stable patients often conveys less short-term risk than preserved EF in the presence of HFpEF, venous congestion, pulmonary hypertension, or elevated biomarkers. Throughout the perioperative course, hemodynamic stability depends less on resting EF than on myocardial reserve, ventricular compliance, volume state, and right ventricular function. All of these may change rapidly under anesthesia, ventilation, fluid shifts, and surgical stress. Focused perioperative echocardiography allows reconciliation of historical imaging with the current clinical state. By systematically integrating relevant information, inappropriate risk inflation in stable patients can be avoided while improving the identification of high-risk phenotypes. Accordingly, perioperative management should prioritize euvolemia, symptom control, and stabilization on GDMT rather than decisions based solely on historical EF values.

Focused perioperative sonography enables anesthesiologists to detect discrepancies between the documented diagnosis and the patient’s current hemodynamic state. For example, it helps to distinguish compensated from decompensated heart failure, recognize venous congestion, and identify right ventricular dysfunction. The ability to integrate these findings with the clinical history and examination enhances diagnostic certainty and supports timely, physiology-guided decision-making.

### Integration of advanced echocardiographic techniques into anesthetic practice

CO measurement, Tissue Doppler Indices of diastolic function, and, where available, strain imaging extends the assessment beyond chamber volumes toward a more comprehensive evaluation of myocardial function (Duncan et al. 2014; Skubas 2009). These modalities allow anesthesiologists to answer questions directly relevant to perioperative care, including preload dependency, ventricular compliance, right-sided contribution to CO, and the presence of subclinical myocardial dysfunction in patients with preserved EF (Abuelkasem et al. 2019; Duncan et al. 2014; Labus et al. 2024).

Routine familiarity with these techniques facilitates collaboration with cardiology and reduces reliance on delayed external imaging. More importantly, it enables the pragmatic integration of echocardiographic information that directly influences perioperative decision-making. As perioperative medicine moves toward physiology-guided care, focused anesthesiologist-performed echocardiography is becoming an essential component of comprehensive cardiovascular risk assessment.

## Gaps in evidence and future directions

Despite growing recognition of the limitations of EF in perioperative risk stratification, several important evidence gaps remain. First, most available data on EF, HFpEF, venous congestion, and advanced imaging have been derived from heterogeneous cohorts or post hoc analyses rather than from trials specifically designed to guide perioperative decision-making. Second, few prospective studies have systematically compared EF-based assessment with multidimensional risk models that integrate functional capacity, congestion, strain imaging, and biomarkers in patients undergoing surgery (Wijeysundera et al. 2018; Cikes and Solomon 2016).

A limitation of the current evidence is that several concepts discussed in this review are derived from non-perioperative populations, particularly critical care cohorts. While these data provide valuable physiological insights, their direct applicability to surgical patients requires careful interpretation.

In addition, the role of advanced echocardiographic techniques in routine perioperative workflows remains incompletely defined. Similar uncertainty applies to biomarker-guided strategies. Future research should therefore focus on prospective, perioperative-specific studies that validate integrated risk models, clarify how imaging and biomarkers should be combined in different patient phenotypes, and determine whether physiology-guided strategies can improve outcomes.

Despite its limitation, EF remains an important clinical parameter in several contexts. In particular severely reduced EF (< 30%) is consistently associated with increased perioperative risk and warrants heightened vigilance (Thompson et al. 2024). Moreover, EF continues to play a central role in heart failure classification and in guiding evidence-based therapies, including pharmacological treatment and device implantation (Heidenreich et al. 2022; Maddox et al. 2024; Russo et al. 2025).

In addition, even after two decades, EF remains the established reference parameter in clinical research, based on the recommendations of the ASE. This role underscores its continued relevance as a standardized and widely accepted metric, particularly in comparative and longitudinal study settings (Gottdiener et al. 2004).

These considerations highlight that EF should not be dismissed but rather interpreted within its appropriate clinical context. In the perioperative setting, its value lies not in isolation, but as part of a broader, physiology-guided assessment.

## Conclusion

EF remains a cornerstone of chronic heart failure management and long-term cardiovascular prognostication, but its uncritical use as a marker of perioperative risk is not supported by contemporary evidence. EF is load-dependent and reflects systolic function only at a single time point. It fails to capture key determinants of perioperative vulnerability, including diastolic dysfunction, venous congestion and right ventricular function. Clinical scenarios such as HFpEF, MR, distributive shock, and chronic compensated heart failure illustrate that EF may either underestimate or overestimate perioperative risk depending on the physiological context.

For anesthesiologists, the implication is not to abandon EF, but to integrate it into a physiology-guided, multidimensional assessment that prioritizes functional capacity, volume status, and clinical stability. Focused perioperative ultrasound, integrated with a thorough clinical history and the targeted use of biomarkers, provides a more coherent and clinically relevant framework for perioperative risk stratification. Moving beyond EF-centric decision-making toward integrated physiological assessment has the potential to improve risk discrimination, avoid unnecessary surgical delay, and support safer, more individualized perioperative care.

## Data Availability

No datasets were generated or analysed during the current study.

## Abbreviations

ACC: American College of Cardiology
ACE: Angiotensin-converting enzyme
AF: Atrial fibrillation
AHA: American Heart Association
AKI: Acute kidney injury
ARNI: Angiotensin receptor-neprilysin inhibitor
ASE: American Society of Echocardiography
CABG: Coronary artery bypass grafting
CO: Cardiac output
CVP: Central venous pressure
DD: Diastolic dysfunction
EACVI: European Association of Cardiovascular Imaging
E/e´: Ratio of early mitral inflow velocity
EF: Ejection fraction
GDMT: Guideline-directed medical therapy
GLS: Global longitudinal strain
HF: Heart failure
HFmrEF: Heart failure with mildly reduced ejection fraction
HFpEF: Heart failure with preserved ejection fraction
HFrEF: Heart failure with reduced ejection fraction
hs-cTnI: High-sensitive cardiac troponin I
hs-cTnT: High-sensitive cardiac troponin T
ICU: Intensive care unit
IVC: Inferior vena cava
IVRT: Isovolumetric relaxation time
LAP: Left atrial pressure
LARS: Left atrial reservoir strain
LAVI: Left atrial volume index
LCOS: Low cardiac output syndrome
LV: Left ventricle
LV-DD: Left ventricular diastolic dysfunction
MACE: Major adverse cardiovascular events
METs: Metabolic equivalents
MINS: Myocardial injury after non-cardiac surgery
MR: Mitral regurgitation
MVR: Mitral valve repair
NT-proBNP: N-terminal pro-B-type natriuretic peptide
NYHA: New York Heart Association
PAD: Peripheral arterial disease
PAP: Pulmonary artery pressure
RCRI: Revised Cardiac Risk Index
RV: Right ventricle
S/D ratio: Systolic/diastolic pulmonary vein flow ratio
SGLT2: Sodium-glucose cotransporter-2
TEE: Transesophageal echocardiography
TR Vmax: Peak tricuspid regurgitation velocity
US: Ultrasound
